# Caterpillar movement mediates spatially local interactions and determines the relationship between population density and contact

**DOI:** 10.1186/s40462-024-00473-x

**Published:** 2024-04-30

**Authors:** Brendan D. Carson, Colin M. Orians, Elizabeth E. Crone

**Affiliations:** 1 Department of Biology, Tufts University, Medford, MA 02155 USA; 2 Department of Evolution and Ecology, University of California, Davis, CA 95616 USA

**Keywords:** Disease ecology, Densovirus, Checkerspot butterfly, Heterogeneity, Clustering, Contact rates, Density dependence, Correlated random walk, Resource availability, Habitat use, Larval stage

## Abstract

**Background:**

While interactions in nature are inherently local, ecological models often assume homogeneity across space, allowing for generalization across systems and greater mathematical tractability. Density-dependent disease models are a prominent example of models that assume homogeneous interactions, leading to the prediction that disease transmission will scale linearly with population density. In this study, we examined how the scale of larval butterfly movement interacts with the resource landscape to influence the relationship between larval contact and population density in the Baltimore checkerspot (*Euphydryas phaeton*). Our study was inspired by the recent discovery of a viral pathogen that is transmitted horizontally among Baltimore checkerspot larvae.

**Methods:**

We used multi-year larvae location data across six Baltimore checkerspot populations in the eastern U.S. to test whether larval nests are spatially clustered. We then integrated these spatial data with larval movement data in different resource contexts to investigate whether heterogeneity in spatially local interactions alters the assumed linear relationship between larval nest density and contact. We used Correlated Random Walk (CRW) models and field observations of larval movement behavior to construct Probability Distribution Functions (PDFs) of larval dispersal, and calculated the overlap in these PDFs to estimate conspecific contact within each population.

**Results:**

We found that all populations exhibited significant spatial clustering in their habitat use. Subsequent larval movement rates were influenced by encounters with host plants and larval age, and under many movement scenarios, the scale of predicted larval movement was not sufficient to allow for the “homogeneous mixing” assumed in density dependent disease models. Therefore, relationships between population density and larval contact were typically non-linear. We also found that observed use of available habitat patches led to significantly greater contact than would occur if habitat use were spatially random.

**Conclusions:**

These findings strongly suggest that incorporating larval movement and spatial variation in larval interactions is critical to modeling disease outcomes in *E. phaeton*. Epidemiological models that assume a linear relationship between population density and larval contact have the potential to underestimate transmission rates, especially in small populations that are already vulnerable to extinction.

**Supplementary Information:**

The online version contains supplementary material available at 10.1186/s40462-024-00473-x.

## Background

Interactions among organisms are inherently local. Nonetheless, many models of ecological dynamics start with the assumption of homogeneous interactions among individuals across space. Incorporating spatially local interactions can change model outcomes in many circumstances (e.g. [59, 62, 67, 68]), but adding complexity also makes models less broadly applicable, somewhat undermining the goal of describing general ecological principles [28]. Furthermore, including spatially explicit interactions is not always necessary to accurately capture system dynamics. For example, after rigorously measuring the effect of local competitive interactions among annual plants to parameterize a dynamic community model, Pacala and Silander [64] found that the scale of seed dispersal was large enough to eliminate any observable effect of local competition over time. In subsequent work, the same authors showed that local interactions were drivers of species distributions among woody plants, where the relatively small scale of dispersal allowed local interactions to predominate [63]. Thus, even though spatial heterogeneity is a near-universal phenomenon, its relative effect depends on the scale of movement in the system of interest.

Density-dependent disease models are a prominent example of the use of the assumption that interactions are spatially homogeneous. The assumption that each host has an equal probability of encountering every other host in a given population [33, 40] results in a linear relationship between population density and conspecific contact rates, and therefore disease transmission [2, 6]. This simplification allows for straightforward estimation of key disease parameters and predictions about disease dynamics [2, 6].

Recently, more attention has been given to the influence of local interactions on disease outcomes. In the well-studied badger-bovine TB system, network clustering created by badger social structure creates disproportionally high levels of contact within clusters, disrupting the relationship between population density and transmission [8, 90, 92]. Similarly, non-linearities between population density and contact can also arise through spatial clustering in habitat use [93]. Clearly, a better understanding of the circumstances under which increasing population density results in more contact can improve our understanding of disease risk, and improve wildlife management decisions [24, 94]. However, understanding when spatially local interactions alter the relationship between density and contact requires work grounded in host movement behavior that spans multiple populations.

The tools available to integrate disease ecology and animal movement vary among taxa. Long-term movement data obtained through geolocated transmitters have been particularly useful for illuminating the host–pathogen contact process in vertebrate taxa such as deer, racoons, zebra, impala, and wildebeest [30, 38, 41, 86]. However, because the majority of mobile species are too small to tag with a transmitter [51], relying on these methods creates a taxonomic bias in our understanding of the processes underlying disease transmission. Advancing our understanding of movement-mediated contact in insects in particular presents an opportunity in disease ecology: many insect pathogens are well-studied, and the recent use of molecular methods have revealed a suite of previously cryptic insect pathogens (Corey and Myers 2004), [1, 29, 34, 37, 56, 57, 73, 79]. Insects play an enormous role in ecological processes, comprising much of the planet’s animal diversity [85, 91]. Moreover, because insect herbivores often feed on plants that have patchy distributions, they present an ideal system for examining the relationship between spatial heterogeneity in habitat use, population density, movement, and conspecific contact.

For insects, there is a long tradition of studying movement by tracking individual animals over short time periods using direct observation, then scaling to long-term dispersal using a correlated random walk (CRW) model [48, 76, 88]. Because herbivorous insects often exhibit CRW movement while searching for suitable food or oviposition sites [12, 16, 39], foraging dispersal can be modeled as a diffusion process. In this study, we use a CRW approach to understand the role of spatially local interactions and larval movement to contact rates in the Baltimore checkerspot butterfly (*Euphydryas phaeton*). Several aspects of *E. phaeton*’s life history make it a particularly tractable study system (e.g., [12, 13, 75, 80–83]. Adult females lay their eggs in batches, which suggests that more frequent interactions may occur among larval groups from adjacent oviposition locations. The disjunct nature of *E. phaeton* populations allows for an examination of the relationship between habitat use, population density, and conspecific contact across independent populations. Finally, a densovirus (JcDV) was recently found in *E. phaeton* populations in New England [57], providing a strong motivation for understanding contact processes in the caterpillar host.

In this study, we integrate observations of caterpillar movement behavior with observed larval nest locations to estimate proportional conspecific contact within different *E. phaeton* populations spanning a range of population sizes and densities. Specifically, we ask: (a) How clustered are Baltimore checkerspot oviposition sites (nests) within available habitat patches?; (b) Given the scale of predicted larval movement, does the observed scale of heterogeneity in habitat use influence predicted larval contact?; and (c) Do observed patterns of habitat use lead to a linear relationship between population density and conspecific contact (i.e. as assumed in simple disease models)?

## Methods

### Study system

*Euphydryas phaeton* occurs throughout much of eastern north America, where it is associated with wetland and old-field habitats. This species is of conservation concern in much of its range, and population sizes are known to fluctuate widely, largely for unknown reasons [22]. This species has one generation per year. Female *E*. *phaeton* lay eggs in clusters in mid-summer, using either *Chelone glabra* or *Plantago lanceolata* as oviposition host plants in the Northeastern United States [9]. Early instar (1st–4th) larvae are gregarious and form silk webs (hereafter “nests”) containing ~ 90–150 sibling larvae [11]. These nests are easily surveyed during late summer and early fall before larval diapause. Larvae overwinter as 4th instars under the leaf litter of their natal nests. After overwintering, post-diapause larvae disperse from their natal sites as non-gregarious foragers, feeding on a suite of plants containing iridoid glycosides [3, 9] and encountering larvae from other nest groups in the process.

Foraging at the postdiapause larval stage likely plays an important role in horizontal disease transmission: viral particles are present in infected larval frass and decomposing cadavers (B. Carson unpubl. data; [57]), and feeding on JcDV-contaminated host plants leads to infection, substantial mortality in post-diapause larvae, and can impact population demographic rates (B. Carson unpubl. data). JcDV can persist on the leaves of food plants for several weeks, but in a separate experiment we found that viral loads decayed by ~ 3 orders of magnitude over a 6-week period, presumably through exposure to UV radiation (B. Carson unpubl. data). Thus in this system, as found in other systems, pathogen transmission is dependent on larvae occupying the same location in space, though not necessarily at the same time [30]. Our study was motivated by the possible implications of nest clustering and spatially local interactions for transmission of JcDV. While understanding drivers of disease was the impetus for this analysis, the nest location data we used were collected before we were aware of JcDV’s presence.

We surveyed *E. phaeton* nests at three sites in Maryland and four sites in Massachusetts over multiple years (Additional file 1: Supplement 1.1, Table S1). All three Maryland populations used *C. glabra* as their host plants. In Massachusetts, the Appleton population used *C. glabra*, the Harvard populations used *C. glabra* and *P. lanceolata*, and the Upton population used *P. lanceolata*. *E. phaeton* habitat is variable in the abundance and distribution of host plant resources, which may influence foraging movement in post-diapause larvae. Movement path data in this study were collected in two habitat contexts: the Upton field population, which had low host plant density, and an old-field site on Tufts’ campus, which had a high host plant (*P. lanceolata*) density.

### Larval nest distributions

Each site was visited multiple times during the larval pre-diapause period in late summer, and nests were surveyed using a sight-resight protocol similar to that detailed in Iles et al. [44], as described by Brown et al. [11]. The surveyor slowly walked over the entirety of the site, and when nests of larvae were found they were recorded and geolocated. This species has one generation per year, and no temporal overlap between the various life-stages. Therefore, the larval nests represent the entire *E. phaeton* population within a site during the year of the survey. Pre-diapause nest locations indicate the starting positions of the mobile post-diapause stage, during which larvae are most susceptible to JcDV transmission. We did not assess the number of larvae in most nests, because this process is invasive and can impact larval survival. However, previous surveys of larval nests show that prior to diapause, nests have ~ 10–200 larvae (median = 50), and about half of these survive to the following spring (Supplement 1.7; see [11, 13]). For the analysis presented in this paper, we use a simple metric of contact that does not depend on nest size (see *Larval contact index*, below).

The habitat available for larval use at each site was determined using field observations of adult butterflies, host plants, and larval nest occurrences (cf. [10]). Briefly, we delineated habitat patch polygons in the field with a GPS, and computed the area within each habitat polygon in ArcGIS. To assess whether each site-years’ larval nest point pattern was more clustered than would be expected by chance, we used a Clark-Evans test with a Cumulative.

Distribution Function (CDF) correction (*spatstat* package; [4]). This Clark-Evans metric (CE-R) assesses the degree to which points patterns are aggregated, randomly, or evenly dispersed within each sites’ habitat polygon compared to a Poisson process point distribution. The nest density of each site-year was calculated by dividing the number of nests found at that site that year by that site’s available habitat area (nests/m^2^). We used nest density as a proxy for butterfly population density, because during late summer the entire population is within pre-diapause nests and can be easily surveyed [11]. The two Harvard populations were separated by a forest, and adult butterflies were occasionally (but not frequently) observed moving between the two patches of meadow during mark-recapture studies [13]. However, these two sites are discontinuous at the scale of larval dispersal (E. Crone, pers. obs) and thus treated as separate populations in our study.

### Larval movement

We collected individual larval movement path data from post-diapause 4th–6th instar larva during May and June 2020. Larval paths were observed in situ at the Upton *E. phaeton* population and ex situ at an old field on Tufts Medford campus. Host plant availability differed substantially between these two sites, and by collecting movement path data under a range of resource densities we hoped to capture a range of larval movement behaviors. 121 paths were observed on 21 separate days, and at the end of each observation we measured each larva’s head capsule width to determine its instar. Larvae released at Tufts for path observation were collected from the Upton population on the preceding day. Each larva was followed for 20 min, and at one-minute intervals we placed a sequentially numbered flag next to the larvae to mark its position. We also noted larva-host plant encounters and feeding bouts, and qualitatively noted larval behavior as they approached host plants. After 20 min, we photographed the path alongside a compass and meter stick.

**Fig. 1:**
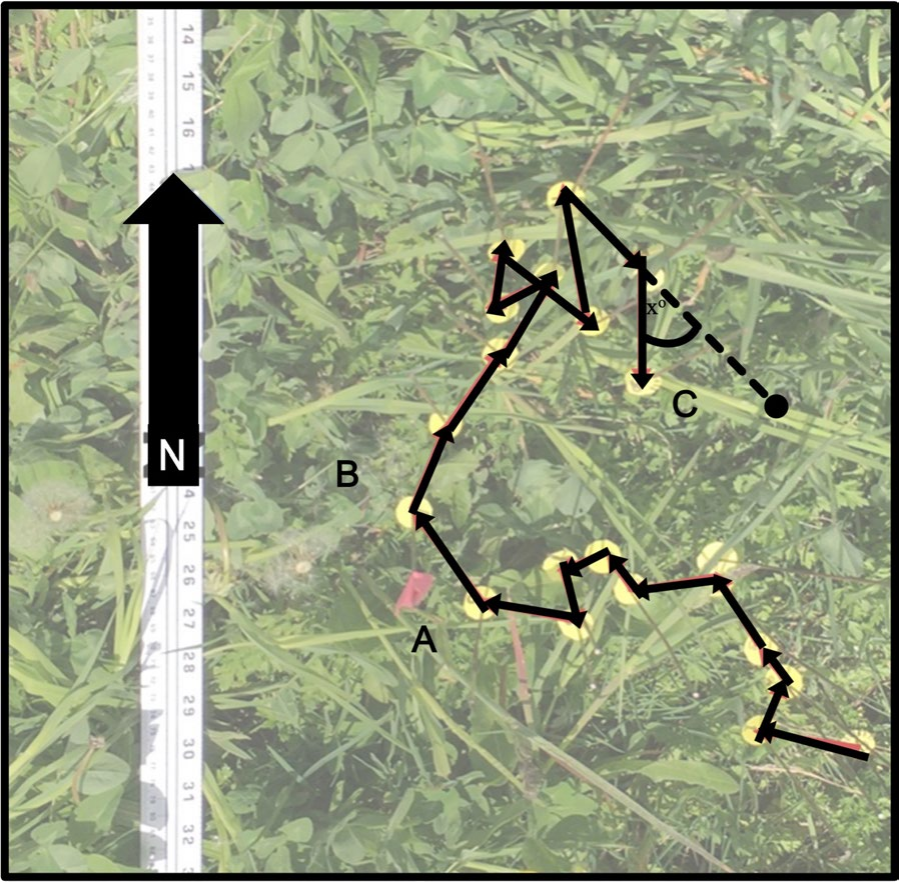
An example of a larval movement path from a 5th instar larva recorded at Tufts’ campus. The larva traversed the entire path over a 20-min period, and each circular disc is on a metal pin that indicates the larva’s location in one-minute intervals. Each movement step is represented by an arrow. In this path, the arrow between points A and B represents the tenth movement step. The arc between the final movement step and the dotted line (C) indicates the turning angle between movement step 18 and movement step 19. N indicates north

Each movement path photo was uploaded to ImageJ FIJI processing software, and we measured the length between each movement step and the turning angle between each move (Fig. 1). These measurements were then used to calculate each movement step’s length (cm), and cosine and sine of the turning angle as in Turchin [89]. Movement path photos were also used to quantify the density of host plants surrounding each larval path. Because individual *P. lanceolata* rosettes variable substantially in their size, we estimated the number of leaves/m^2^.

We limited our statistical analyses to movement paths that contained at least 4 movement steps (Upton *n* = 31; Tufts *n* = 58). We used linear mixed effects models in R (lme package; [5]) with date and individual larvae ID as random factors to investigate whether the movement path parameters of step length, cosine and sine of the turning angle were influenced by larval instar or data collection site. Prior to conducting analyses, we transformed the cosines and sines of the turning angles prior to analysis by dividing by two and adding 0.5 [77], and examined the residuals of each independent variable for normality. We conducted marginal hypothesis testing to evaluate model terms and dropped non-significant interactions (Crawley 2012). Terms in the final model were evaluated using marginal likelihood ratio tests, implemented with the car,;Anova() function in R. The variables in the final model for each movement parameter were then used to generate the diffusion coefficients as described below.

As a secondary analysis, we evaluated whether host plant encounters altered larval movement behavior. We tested each movement parameter using linear mixed effects models with date and larvae ID as random factors, and a fixed effect of whether the larvae had yet encountered a host plant in each movement trial. We limited this analysis to the Tufts dataset, because the host plant encounter rate at Upton was too low to provide sufficient data for statistical inference (15 out of 356 movement steps at Upton occurred after host plant encounters, vs. 115 out of 507 movement steps at Tufts).

We compared the CRW-predicted and observed larval displacements (Additional file 1: Figure S1) and found the CRW diffusion approximation, although slightly lower, was a reasonable approximation of larval displacement and therefore an appropriate model for *E. phaeton* larval movement (Supplement 1.3). Because larval instar and path observation site were both significant predictors of movement step length (see *Results*), we generated diffusion coefficients using site specific step length parameters:

1 $$D = \left( {m_{2} + \left( {2m_{1}^{2} } \right)*\left( {\frac{\Psi }{{1 - \Psi }}} \right)} \right)/(4t)$$

where D is the diffusion coefficient, $\Psi$ is the mean cosine of the turning angle, ${m}_{1}$ is the site and instar-specific mean step length and ${m}_{2}$ is the site and instar specific mean squared step length and *t* is the time spent in moving in a given instar [89],Table 2).

### Larval contact index

We used the instar-specific diffusion coefficients to generate two-dimensional Gaussian Probability Distribution Functions (PDF) centered on each larval nest site (Additional file 1: Supplement 1.4, Figure S2). The PDF’s standard deviation is $\sqrt{2tD}$ where *t* is the time spent dispersing during a given post-diapause instar (estimated from field data; Supplement 1.5) and *D* is the instar and rate-specific diffusion coefficient calculated above. The PDF for 4th, 5th and 6th instars accounted for dispersal that would have occurred during previous post-diapause instars by using an average diffusion coefficient weighted by mean number of days spent in each instar (estimated from experimental data; Supplement 1.6). To estimate the population-wide proportional contact for a given year, we generated a metric to describe likely contact among nests by quantifying the amount of overlap between each larval nest’s PDF using Schoener’s Index [74, 78]:

2 $$1-\frac{1}{2}\sum_{i=1}^{n}|p(x{)}_{i}-p(y{)}_{i}|$$

where p(x) and p(y) represent the two PDFs, and index values of 0 and 1 indicate no overlap or complete overlap, respectively.

We used the *fMultivar* package in R [95] to simulate a grid of 1 × 1 m cells (*i*), projected each PDF on the grid so that each cell’s z value represented the probability associated with each PDF. As a simple metric of relative contact rates for each site-year, we computed the Schoener’s overlap between every pair of larval nests at each instar stage (see Supplement 1.4). Then we summed each nest’s total overlap. A site-year’s contact index is the average of each of these nest-level contact estimates. This analysis was performed once using movement parameters estimated from Upton movement paths and again using Tufts movement parameters.

After calculating the mean contact index for each site-year, we evaluated site-year means of Schoener’s overlap index using mixed effects models (lme4 package, [5]) with movement rate (Upton vs. Tufts), larval instar, number of nests, nest density, and the Clark-Evans clustering metric as fixed-effect predictors, and site as a random factor. Because nest density was calculated using nest number, these factors are collinear and should not be included in the same model [96]. Therefore, we first constructed two separate full-interaction models using each of these parameters and compared them using delta AIC. We then removed nonsignificant interactions from the best model (nest density vs. nest number). Terms in the final model were evaluated using type II marginal likelihood ratio tests, implemented with the car::Anova() function in R.

To investigate the effect of observed spatial clustering on larval contact, we generated 20 replicates of simulated Poisson-process point patterns at the same densities as observed nests for each site-year. We combined the overlap data resulting from these simulated nest distributions with overlap data from observed nest distributions and then used a mixed effect model to test for the effect of movement rate, larval instar, and point pattern source (observed or simulated), with site as a random factor using the model selection approach described above.

## Results

### Larval nest distributions

**Fig. 2:**
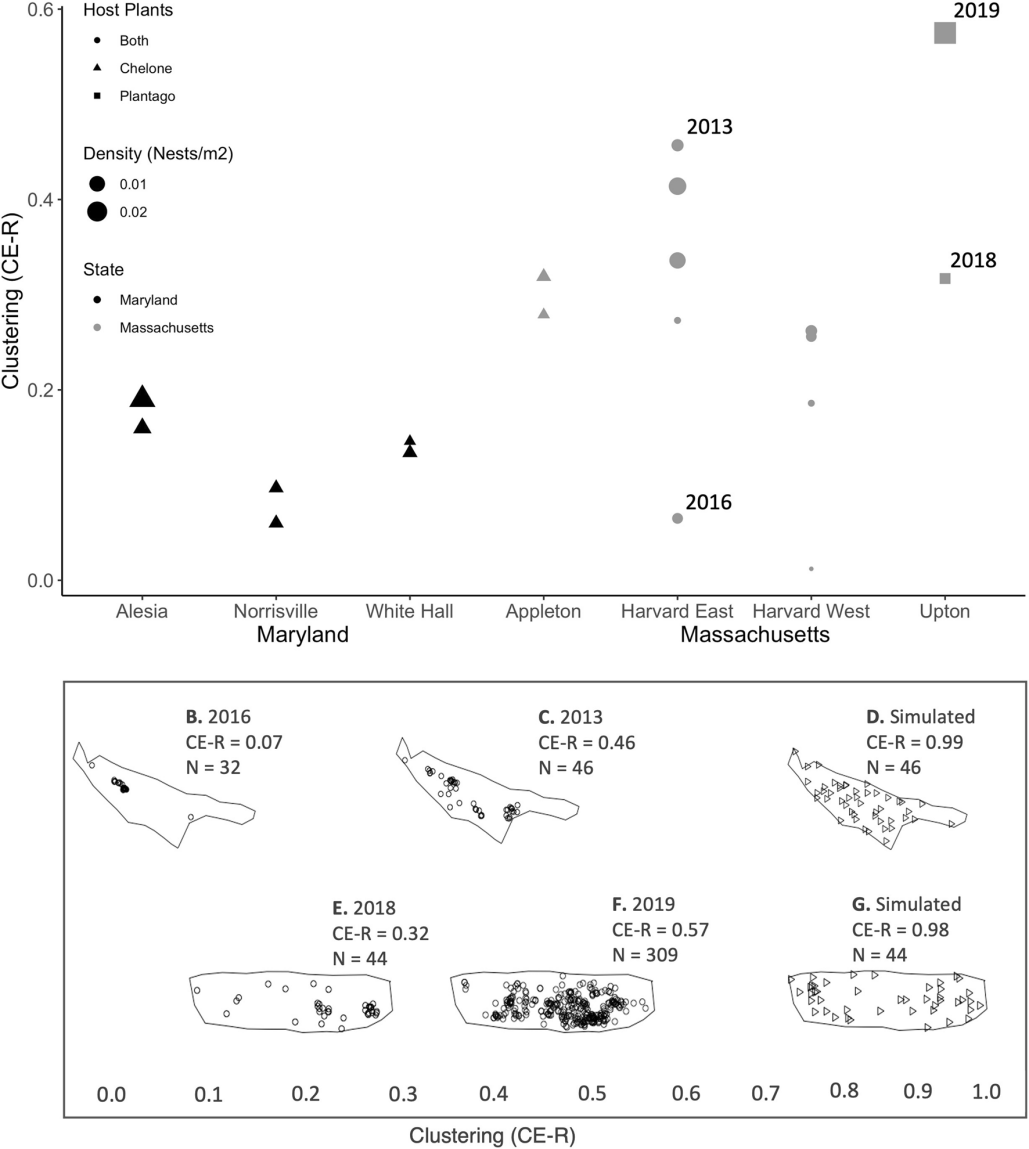
Top panel:** A** Spatial clustering within the *E. phaeton* populations surveyed in this study. The y axis depicts the Clark Evans clustering metric (CE-R), in which values closer to zero indicate a higher degree of clustering. Each point denotes a year of survey: all sites were surveyed in 2018 and 2019, and the Harvard sites were also surveyed from 2013 to 2016. Figure legend indicates the state of origin, nest density (nests/m^2^), and oviposition host plants used at the site. The points corresponding to sites-years in bottom panel are indicated with year labels. Bottom panel: An example of nest spatial distributions corresponding to different CE-R values: **B** The observed nest distribution of the Harvard East population in 2016 (n = 32); **C** The observed nest distribution of the Harvard East population in 2013 (n = 46); **D** A simulated Poisson point-pattern within the Harvard East habitat polygon (n = 46); **E** The observed nest distribution of the Upton population in 2018; **F** The observed nest distribution of the Upton population in 2019; **G** A simulated Poisson-process point pattern within the Upton habitat polygon (n = 44)

The number of Baltimore checkerspot nests detected ranged from a low of four (Harvard East in 2019) to a high of 309 (Upton in 2019; Fig. 1F; Additional file 1: Table S1). Available habitat ranged from 1190 m^2^ (Alesia) to 21,821 m^2^ (Harvard West; Additional file 1: Supplement 1.2, Table S1). The Clark-Evans R values for all site-years were below 1.0, suggesting that nests were more clustered than expected by chance (Fig. 2). Larval nest distributions in fourteen of the 20 site-years were significantly more clustered within available habitat than expected by chance (Additional file 1: Table S1); the remaining six sites-years had too few observed nests to conduct a significance test using the Clark-Evans method.

### Larval movement

The best model of step length included the covariates of larval instar and observation site without interactions. Movement lengths ranged from 0.6 to 58.5 cm per one minute step interval, with larvae traversing Euclidian distances between 0.1 and 9.5 m over the 20-min observation period. Movement step lengths increased with larval ontogeny (*χ*^2^ = 4.95, *df* = 1, *P* = 0.03), and Upton movement paths had longer step lengths than those observed at the Tufts campus site (*χ*^2^ = 26.81, *df* = 1 *P* < 0.001) across all instars. There was variability in step length between individual larvae (larvae ID random effect *sd* = 3.4). The sine and cosine of movement path turning angles did not differ as a function of any predictor variables. Movement parameter means and confidence intervals are provided in Table 1, and the results of the likelihood ratio tests used to evaluate each movement parameter can be found in Supplement 1.2 (Additional file 1: Table S2).

**Table 1:** Mean movement parameters estimated from movement path data

Parameter*	Mean	Lower CL	Upper CL	SE	Degrees of Freedom* (df)*
Sine of turning angle	0.01	− 0.05	0.06	0.03	15.9
Cosine of turning angle	0.32	0.19	0.45	0.06	18.4
Step time (s)	102	92	113	4.90	15.2
Step length (cm)					
Instar 4 Upton	6.69	0.00	14.9	4.00	26.08
Instar 5 Upton	12.03	6.83	17.2	2.17	6.51
Instar 6 Upton	12.01	7.91	16.10	1.72	6.76
Instar 4 Tufts	4.03	3.03	5.02	0.45	10.50
Instar 5 Tufts	4.24	3.35	5.13	0.42	16.60
Instar 6 Tufts	7.62	5.96	9.28	0.79	21.50
Step Length^2^ (cm)					
Instar 4 Length^2^ Upton	113	0.00	391	135.40	27.20
Instar 5 Length^2^ Upton	267	86.60	447	67.30	4.40
Instar 6 Length^2^ Upton	201	74.00	327	52.10	6.19
Instar 4 Length^2^ Tufts	22.50	4.59	40.50	8.06	10.00
Instar 5 Length^2^ Tufts	25.30	8.96	41.70	7.81	18.20
Instar 6 Length^2^ Tufts	103.30	71.25	135.30	15.90	15.90
Diffusion coefficient (D)**					
Instar 4 D Upton	0.38	0.14	1.11		
Instar 5 D Upton	0.99	0.45	1.43		
Instar 6 D Upton	0.82	0.49	1.35		
Instar 4 D Tufts	0.09	0.07	0.15		
Instar 5 D Tufts	0.10	0.08	0.16		
Instar 6 D Tufts	0.39	0.19	0.46		

*Larval movement lmer models described in methods

**Estimated using Eq. 5.3 from Turchin [89]: $D= {(m}_{2}+\left(2{m}_{1}^{2}\right)*\left(\frac{\Psi }{1-\Psi }\right))/(4t)$

*P. lanceolata* was less abundant at Upton than Tufts (means of 15.9 (95% CI 0–50.8) leaves/m^2^ and 102.7 (95% CI 82.2–123.2) leaves/m^2^ respectively). In our analysis of the Tufts campus data, larvae decreased step lengths (*χ*^2^ = 38.19, *df* = 1, *P* < 0.001) and increased time between movement steps (*χ*^2^ = 15.27, *df* = 1, *P* < 0.001) after their first encounter with a host plant.

### Larval contact index

**Fig. 3:**
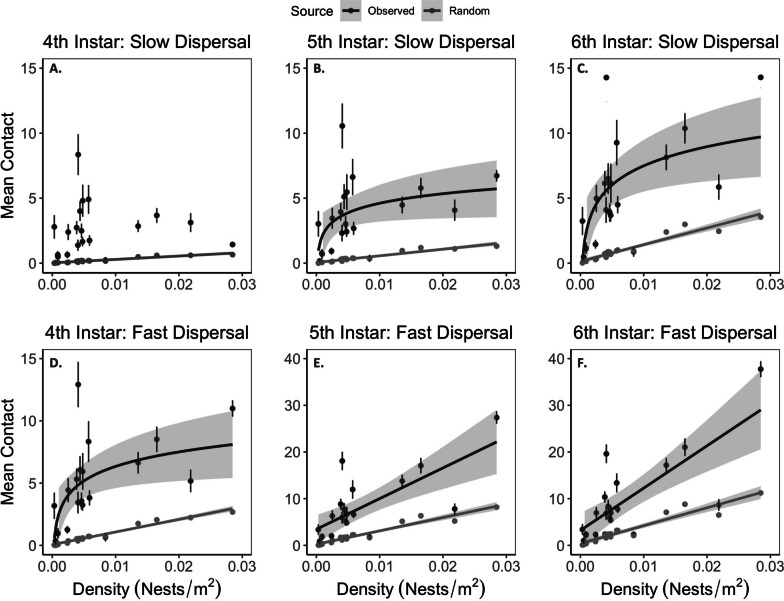
The mean per-nest estimated contact for observed nest distributions (black) and simulated random nest distributions (grey) plotted against nest density. The top three panels (**A–C**) show expected overlap following 4th, 5th, and 6th instar dispersal using movement parameters from Tufts campus (i.e. slow) movement path data, and the bottom three panels (**D–F**) show expected mean overlap following 4th, 5th, and 6th instar dispersal using movement parameters from Upton (i.e. fast) data. We competed linear (Contact ~ Density) and log-linear (Contact ~ log(Density)) fit lines using AIC. Best fit lines are included where they significantly fit the data (*p* < .05). Error estimates of points and best fit lines show 95% confidence intervals

**Fig. 4:**
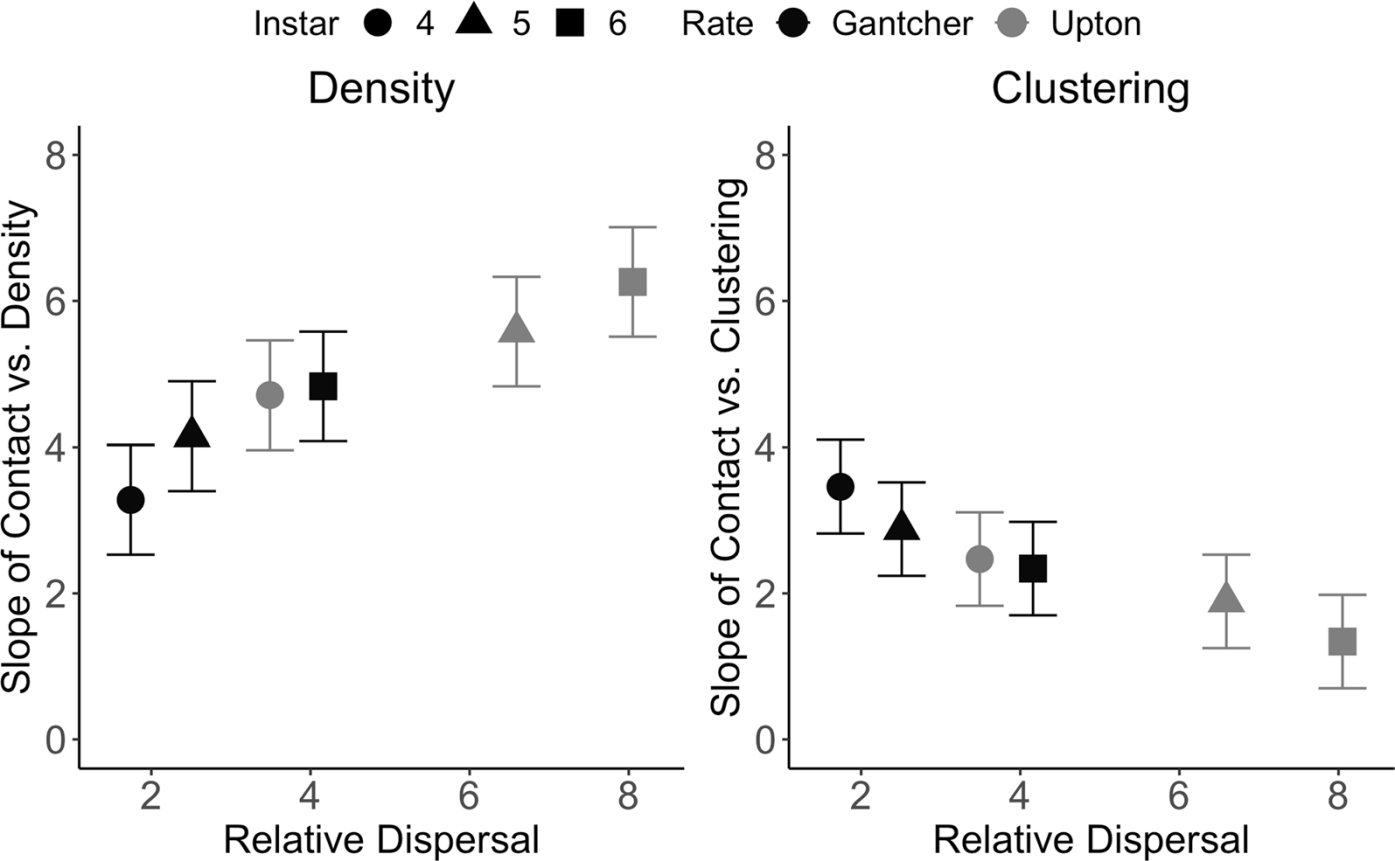
The slopes of Contact vs. Density and Contact vs. Clustering in response to the degree of larval dispersal, calculated from the statistical model shown in Table 2. The x-axis is the standard deviation of the probability distribution of larval locations at the end of each instar

Larval density was a better predictor of contact than population size (ΔAIC = 146.2). In this model, density, clustering, movement rate, instar, and 3-way interactions between Density, Clustering and Instar and Density, Clustering, and Rate significantly affected contact (Table 2). The site-wide contact index increased with dispersal rate and larval instar stage (significant positive main effects, Table 2; Fig. 3). The contact index also increased with nest density and clustering (significant positive main effects, Table 2). Although we did not attempt to interpret each interaction, one clear pattern was that the importance of nest density (i.e. slope of contact vs. nest density) increased with greater dispersal (Fig. 4A). Conversely, the importance of clustering (i.e. slope of nest clustering vs. nest clustering) decreased with greater dispersal (Fig. 4B).

**Table 2:** Summary statistics from analysis of contact index^1^

Random effects	Variance	SD
Site	16.80	4.09
Residual	6.46	2.54

Marginal hypothesis tests	Chisq	*Df*	*P* value
Density	181.33	1	< 0.001
Clustering	33.71	1	< 0.001
Rate	57.94	1	< 0.001
Instar	46.15	2	< 0.001
Density:Clustering	0.05	1	0.831
Density:Rate	22.36	1	< 0.001
Clustering:Rate	5.51	1	0.016
Density:Instar	18.07	2	< 0.001
Clustering:Instar	4.44	2	0.108
Density:Clustering:Rate	11.29	1	< 0.001
Density:Clustering:Instar	9.52	2	0.008
*Density:Rate:Instar*	2.78	2	0.249
*Clustering:Rate:Instar*	0.83	2	0.660
*Density:Clustering:Rate:Instar*	1.74	2	0.419

^1^Non-significant interactions from the full model are shown in italics. Other statistics are from the reduced model with these terms removed

The contact index estimated from observed nest locations also differed significantly from randomly distributed nests. The mean contact index across all dispersal scenarios was 4.42 (95% CI 3.16–5.68) for observed point patterns and 1.07 (95% CI 0–2.32) for simulated random point patterns. Contact indices were always higher for observed than random nest locations (*χ*^2^ = 35.44, *df* = 1, *P* < 0.001), and, as expected, increased with larval instar (*χ*^2^ = 112.74, *df* = 2, *P* < 0.001), and movement rate (*χ*^2^ = 10.15, *df* = 1, *P* = 0.002). This difference between observed and random nest locations was more important in conditions with less movement, as reflected by a significant interaction between nest data source (observed vs simulated) and instar (*χ*^2^ = 4.19, *df* = 2, *P* < 0.001).

## Discussion

*E. phaeton* larval nests were spatially clustered enough to affect contact among larvae. Under most conditions, spatially local interactions disrupted the linear relationship between population density and contact. These results are intuitive: higher population density only results in more contact when individuals in a population move enough to encounter each other. Conversely, when movement is low, only near neighbors come into contact, and spatial structuring strongly influences contact rates. Spatially local interactions can influence ecological processes including disease, predation, and resource competition [28], and other authors have also found that movement is a key determinate of how local interactions scale up to population-level effects (eg. [52, 54, 64]). Our results further illustrate how understanding the relative scales of habitat heterogeneity and animal movement is critical to determining how spatially local interactions impact ecological dynamics.

In our study, heterogeneity in spatially local interactions resulted from initial clustering of larval aggregations within available habitat patches. Our larval nest surveys demonstrate that the degree of habitat-use heterogeneity can vary widely between different populations, suggesting that the relative importance of spatially local interactions is context-dependent. We did not attempt to explain the underlying cause of clustered nest locations, but many factors can influence female butterfly oviposition, including floral resource distribution [58], host-plant abundance, and intraspecific host-plant variability [42, 69, 72]. Smaller *E. phaeton* populations tended to exhibit the highest degree of clustering, which may be tied to the use of highest-quality habitat patches by fewer individuals. Anecdotally, when the Harvard population was declining from 2016 to 2019 (cf. [22]), we found nests in a relatively small proportion of the available habitat, despite the widespread availability of host plants. More generally, numerous past studies have evaluated the relationship between maternal oviposition preference and offspring performance [35, 36, 53, 87]. Most of these studies have focused on offspring performance in relation to food quality; our results suggest that oviposition preference also affects the extent of spatial clustering, and therefore the frequency of larval interactions.

The relative importance of spatial clustering in *E. phaeton* was determined by the scale of larval movement throughout their ontogeny. Our inference about movement is based on a simple model of diffusion coefficients estimated from correlated random walks (CRW). These models have a long history of use with insects [21, 25, 48–50], especially butterflies (e.g., [12, 13, 32, 48, 76, 88], and at least one previous study used CRW models to evaluate larval butterfly movement (the Oregon silverspot, [7]). Our goal was to obtain a rough estimate of the scale of movement in relation to clustering, and, as such, this model is appropriate for our study. However, CRW, like any model, is a simplification of real movement, and may be especially prone to underestimating long-distance movement [45, 70]. One incidental result of our study was variation among parameters estimated for individual animals. Exploring the source of this variation would be a valuable next step, because persistent behavioral differences could lead some individuals to act as “superspreaders” (cf. [84]). Another interesting step would be to build spatially explicit individual based models [86] or network models [92] of this system once we know more about the biology of JcDV transmission, its effects on larval survival, and the possibility of shared reservoir hosts (e.g., [56, 57, 60, 71]).

We estimated an index of contact based on the overlap of larval probability distribution functions (PDFs) following each stage of larval dispersal. In addition to the CRW model constructed from movement path data, the PDFs incorporated the amount of time larvae spent moving, as well as estimates of how long post-diapause larvae spent in each instar. The former estimates were based on field observations, while instar duration was measured in a lab setting. Our lab conditions did not incorporate the temperature variation found in nature, and this could have influenced larval development. In a separate field experiment on Tufts campus, the development time from emergence to pupation ranged from 38 to 59 days in 171 butterflies over two years. The mean time from 4th instar emergence from diapause to pupation in a lab (42 days) was within this range, but the time in each instar may be skewed in the field: cool temperatures early in the spring would lengthen the time spent in the 4th instar, and warmer temperatures later in the season could mean less time spent in later instars. However, our finding that variation in larval movement was the main driver of contact is robust to this amount of variability in development time.

In our study, Baltimore checkerspot movement rates depended on habitat context: larvae at the Tufts site moved more slowly after they encountered host plants. Larvae also exhibited substantially greater movement rates at Upton than Tufts, and these differences may have been caused by the resource landscape at each site. Specifically, sites with a high density of host plants, and thus slower larval movement, are expected to result in stronger local interactions. Many animals move more slowly when they encounter resources, e.g., adult butterflies moving through host plant patches [23, 32, 76], lady beetles near aphid prey [49], and wading birds in relation to tidal flat invertebrates (Dias et al. 2009). From this we would predict that in a disease context, slower movement in resource-dense patches will facilitate pathogen accumulation in resource hotspots [30, 41, 65] but minimize population-wide transmission. Conversely, lower resource density will lead to greater movement throughout a habitat, resulting in a more evenly distributed disease load in the environment and among individuals. This interplay between resource density and movement could also contribute to a feedback between population density and larval contact: higher population densities can lead to faster resource consumption, subsequently resulting in faster movement as larvae search for additional food sources.

The site-based differences we observed in larval movement influenced the shape of the relationship between population density and conspecific contact. In conditions with slower movement, the relationship between density and contact was relatively weak, and showed a decelerating (asymptotic) trend. The relationship between contact and density directly influences disease transmission patterns [2, 40, 65], and analyses of wildlife epidemiological data often find that an asymptotic relationship between population density and transmission best describes observed disease spread [40]. While an asymptotic pattern could result from individual variation in disease susceptibility [14, 65] or vector-based transmission [6], non-linear relationships between contact and population density could also drive these results [30, 38, 40, 90, 92]. Our work demonstrates that spatially heterogeneous local interactions can similarly create non-linearities between contact and population density when movement is limited. With greater larval dispersal, contact in our study was more strongly proportional to population density. These high-movement scenarios approach the homogenous mixing assumed by density-dependent disease models [27, 33], resulting in a linear relationship between density and contact. However, even when the relationship between contact and nest density was linear, our index of contact based on observed nest distributions was always higher than the index calculated for a random distribution of nests on the landscape.

Our study was motivated by the possible implications of nest clustering and spatially local interactions for transmission of a viral disease, JcDV [57]. Because the minimum population density required to sustain pathogen spread (S_R0_) is directly proportional to the estimated transmission rate β, and β is proportional to the contact rate, the increase in contact caused by clustering could lower the population threshold allowing for disease persistence [27, 40, 55]. Fofana and Hurford [33] show that changes in host movement can influence the rate of disease spread in a hypothetical host–pathogen system, but introducing spatial heterogeneity is necessary to alter SR_0_. If a threshold density is estimated assuming a homogenous host distribution, it may overestimate the minimum population that will sustain pathogen spread (Connor and Miller, 2004). This last point is important, because small populations that appear to be below the threshold required to sustain transmission are already vulnerable to extinction from Allee effects and stochastic perturbations [24, 26, 66].

In the Baltimore checkerspot-JcDV system, spatial clustering of larval nests is likely to affect disease transmission, both qualitatively and quantitatively. Our findings also suggest that different populations of the same species can have different relationships between transmission and density. Disease loads differ among Baltimore checkerspot populations in our region [57], and an exciting next step would be to determine if this variation is associated with spatial clustering of habitat, resource density, or larval movement dynamics. Developing a broader framework of how abiotic and biotic determinates influence habitat use and subsequent disease transmission would greatly advance our ability to predict outcomes in specific wildlife populations. Finally, this study reinforces that homogeneous mixing models of local transmission between individual hosts are best applied at scales determined by host movement, rather than defining a ‘patch’ by the amount of contiguous habitat. More generally, our work shows the power of linking spatial pattern and animal movement to inform the conditions under which spatially local interactions are likely to drive ecological dynamics.

### Supplementary Information

**Additional file 1**. Supplemental information supporting the main manuscript, including site desriptions, an assessment of the correlated random walk model, population-level surveys of larval movement behavior, and data linking nest numbers to total population size.

## Data Availability

The datasets and R code used for these analyses are available from the corresponding author on reasonable request.
