# Supplementary material for: Caterpillar movement mediates spatially local interactions and determines the relationship between population density and contact
Source: Mov Ecol. 2024 Apr 30;12:34. doi: 10.1186/s40462-024-00473-x (PMC11061915; doi:10.1186/s40462-024-00473-x)
Supplement: Supplementary file 1 — Additional file 1. Supplemental information supporting the main manuscript, including site desriptions, an assessment of the correlated random walk model, population-level surveys of larval movement behavior, and data linking nest numbers to total population size. [file 40462_2024_473_MOESM1_ESM.docx]

***SUPPLEMENTAL MATERIALS***

***Section 1.1***

***Larval Nest Distributions***

**Supplemental Table 1.** Larval Nest Surveys Spatial Analyses

| **Maryland** | | | | | | |
| --- | --- | --- | --- | --- | --- | --- |
| **Site** | **Year** | **Host Plant** | **Nests** | **Area (m^2^)** | **CE-R*** | **p-value^**^** |
| Alesia | 2018 | Turtlehead | 10 | 1190 | 0.16 | NA**^+^** |
| Alesia | 2019 | Turtlehead | 26 | 1190 | 0.09 | < .001 |
| Norrisville | 2018 | Turtlehead | 25 | 5198 | 0.06 | < .001 |
| Norrisville | 2019 | Turtlehead | 24 | 5198 | 0.09 | < .001 |
| White Hall | 2018 | Turtlehead | 24 | 5028 | 0.13 | < .001 |
| White Hall | 2019 | Turtlehead | 12 | 5028 | 0.15 | NA |
| **Massachusetts** | | | | | | |
| Appleton | 2018 | Turtlehead | 35 | 13906 | 0.28 | < .001 |
| Appleton | 2019 | Turtlehead | 61 | 13906 | 0.32 | < .001 |
| Harvard East | 2013 | Both | 46 | 7817 | 0.45 | < .001 |
| Harvard East | 2014 | Both | 106 | 7817 | 0.34 | < .001 |
| Harvard East | 2015 | Both | 129 | 7817 | 0.41 | < .001 |
| Harvard East | 2016 | Both | 32 | 7817 | 0.07 | < .001 |
| Harvard East | 2018 | Both | 7 | 7817 | 0.27 | NA |
| Harvard East | 2019 | Both | 4 | 7817 | 0.33 | NA |
| Harvard West | 2013 | Both | 19 | 21821 | 0.19 | NA |
| Harvard West | 2014 | Both | 84 | 21821 | 0.26 | < .001 |
| Harvard West | 2015 | Both | 112 | 21821 | 0.26 | < .001 |
| Harvard West | 2016 | Both | 7 | 21821 | 0.01 | NA |
| Upton | 2018 | Plantain | 44 | 10854 | 0.32 | < .001 |
| Upton | 2019 | Plantain | 309 | 10854 | 0.58 | < .001 |

*Clark-Evans R metric; smaller numbers indicate more clustering

**^**^** p-value for test that CE-R was significantly different from 1.0

**^+^** NAs indicate that the number of nests was too small to conduct a significance test on CE-R

***Section 1.2***

***Movement Parameter Analysis Likelihood Ratio Results***

**Supplemental Table 2.** Likelihood Ratio Test (car::Anova) results from full model used in model selection to determine which parameters needed site and instar-specific values in diffusion calculations

| **Movement Parameter** | **Model** | **Factor** | **Chisq** | **Df** | **P** |
| --- | --- | --- | --- | --- | --- |
| Step Length | Length ~ Instar*Site | Instar | 4.78 | 1 | **0.03** |
|  |  | Site | 32.18 | 1 | **<0.001** |
|  |  | Instar:Site | 0.002 | 1 | 0.96 |
| Step Length^2^ | Length^2^~ Instar*Site | Instar | 0.97 | 1 | 0.33^*^ |
|  |  | Site | 28.36 | 1 | **<0.001** |
|  |  | Instar:Site | 0.58 | 1 | 0.44 |
| Scaled Cosine | Cosine ~ Instar*Site | Instar | 0.51 | 1 | 0.48 |
|  |  | Site | 2.01 | 1 | 0.16 |
|  |  | Instar:Site | 1.43 | 1 | 0.23 |
| Scaled Sine | Sine ~ Instar*Site | Instar | 0.51 | 1 | 0.48 |
|  |  | Site | 2.01 | 1 | 0.48 |
|  |  | Instar:Site | 1.43 | 1 | 0.23 |
| Step Time | Time ~ Instar*Site | Instar | 0.03 | 1 | 0.87 |
|  |  | Site | 0.25 | 1 | 0.62 |
|  |  | Instar:Site | 1.92 | 1 | 0.17 |

*******We included instar-specific Step Length^2^ values in our diffusion estimates because this variable was derived from Step Length, which was significantly different between each instar.

***Section 1.3***

**Assessment of Correlated Random Walk**

***Methods:***

We assessed whether larval movement was directionally biased by testing the null hypothesis that the sine of the turning angle was 0 (i.e. there were equal right and left turns). We used linear mixed effects models in R (lme package; Bates et al., 2015) with date and individual larvae ID as random factors to investigate whether the sine of movement path turning angles was influenced by larval instar or data collection site (as described in main text). We assessed significance with marginal likelihood ratio tests, implemented with the car;Anova() function in R. To test whether a CRW is an appropriate model for larval movement, we compared the net squared displacement (NSD) from field observations to CRW model predictions. We first calculated the NSD between each possible step interval for each observed movement path and used mixed effects models to estimate the mean observed NSD for each larval instar. We then used mean movement parameters to estimate the expected NSD (Turchin 1998; his Eq. 5.2):

| $\boldsymbol{NSD=}\boldsymbol{n*m}_{\boldsymbol{2}}\boldsymbol{+}\left( \boldsymbol{2}\boldsymbol{m}_{\boldsymbol{1}}^{\boldsymbol{2}} \right)\boldsymbol{*}\left( \frac{\boldsymbol{\Psi}}{\boldsymbol{1-\Psi}} \right)\boldsymbol{*}\left( \boldsymbol{n-}\left( \boldsymbol{1-}\frac{\boldsymbol{\Psi}^{\boldsymbol{n}}}{\boldsymbol{1-\Psi}} \right) \right)$ | S. Eq. 1 |
| --- | --- |

where *n* is the number of movement steps, *m*_1_ is the mean movement step length, *m*_2_ is the mean squared movement step length, and $\Psi$ is the mean turning angle cosine. Finally, we plotted the NSDs from CRW model predictions and observed NSDs together to assess whether the observed NSD fell within the 95% confidence intervals of the CRW predictions.

***Results:***

The scaled sine of larval turning angles had a mean distributed around 0 (95% CI -0.05—0.06; Main Text Table 1), and was not influenced by movement path observation site (*χ*^2^ =0.01, *df* = 1 *P*=0.92) or larval instar (*χ*^2^ =0.22, *df* = 1 *P*=0.64). The observed NSDs fell within the confidence intervals of predicted displacements for the Upton data, but were slightly higher than the predicted displacements for the Gantcher data (Supplemental Figure 1). While slighty higher, in both cases the CRW prediction provide a reasonable approximation of *Euphydras phaeton* larval movement.


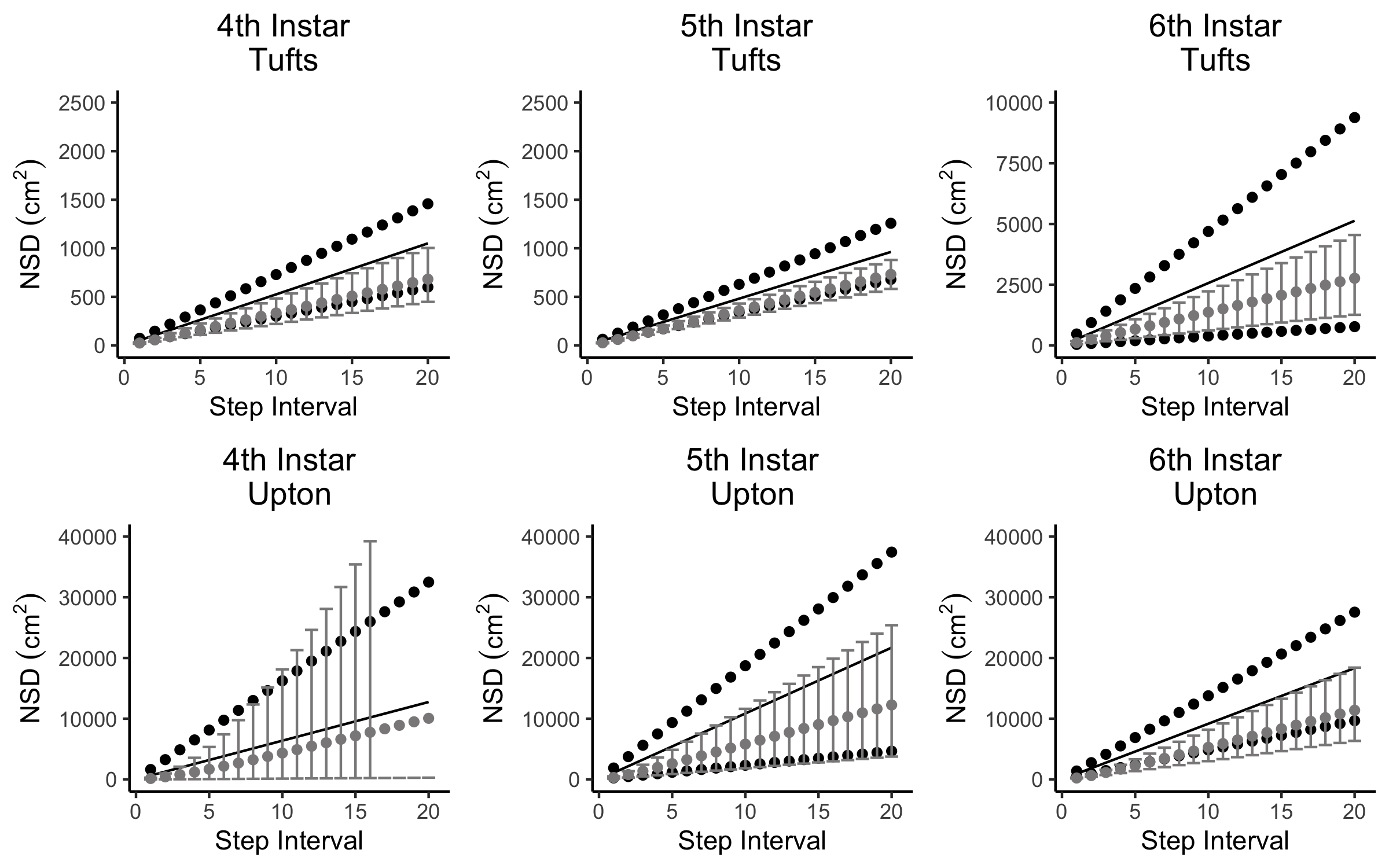


**Supplemental Figure 1.** The Net Squared Displacements (NSD) from observed larval movement paths (black line with black points showing 95% CI) and Correlated Random Walk (CRW) model predictions (grey, with error bars showing 95% CI). Movement parameters are calculated for 4^th^-6^th^ larval instars at Tufts and Upton observation sites.

***Section 1.4***

***Probability Distribution Functions (PDFs) and Schoener’s Overlap***


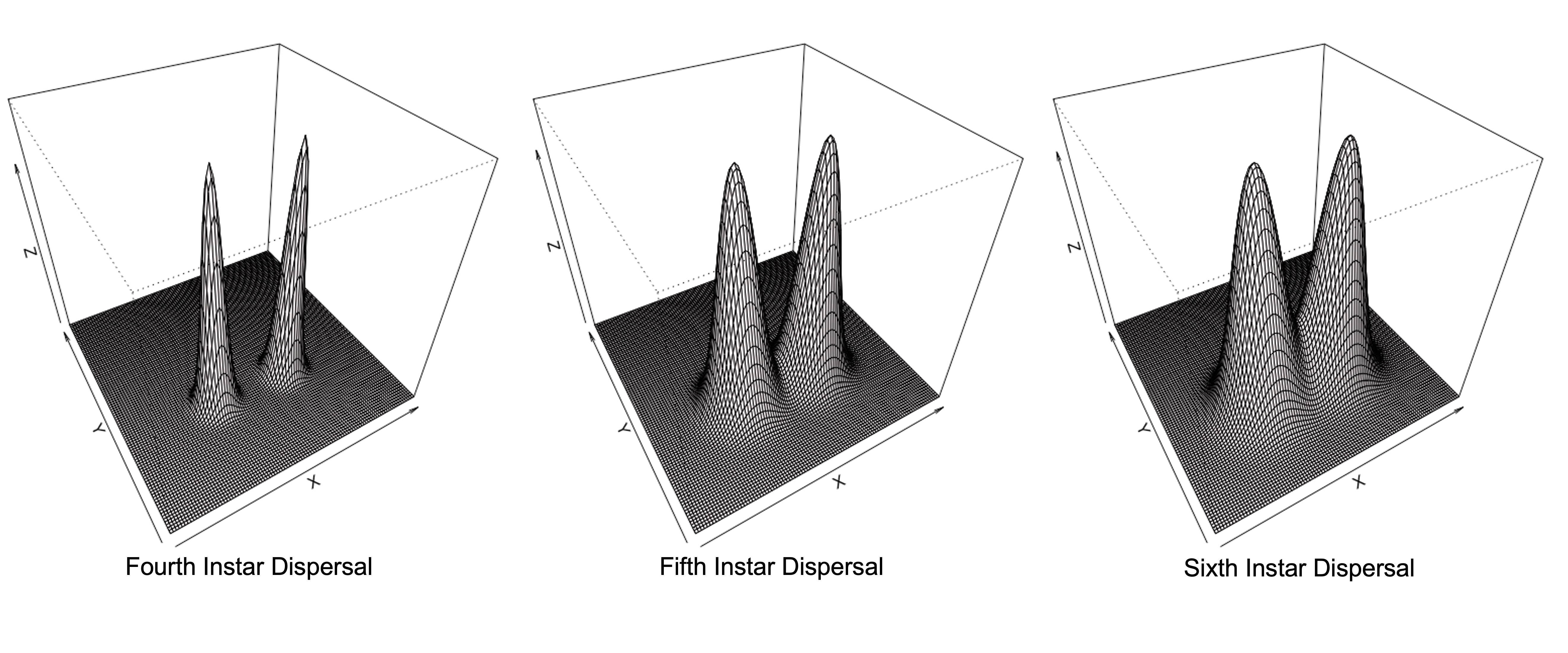


**Supplemental Figure 2.** The Probability Distribution Functions (PDFs) for two larval nests located 30 m apart after each instar’s dispersal period. These PDFs were created using movement parameters from Upton larval movement paths. This figure is given as a visual example of the PDFs used to calculate pairwise Schoener’s overlap in the main text. The overlap index values associated with each figure from left to right are 1.9 e-5, 0.02, and 0.06.

The Schoener’s index is not a count of larval contact events, but instead a relative metric of overlap of the instar and dispersal rate-specific PDFs associated with each nest location. We expect that the overlap indices are proportional to the number of larval contact events within a given site-year, but extrapolating these indices to precise numbers of larval contact events is not possible.

To help understand the process of calculating the Schoener’s overlap metric that we used for a contact metric, here is an example using a population with 4 hypothetical nests in different relative positions. Each nest has 3 pairwise interactions with other nests in the population, and there are 6 unique pairwise interactions across the population. If each nest overlapped completely (i.e. same gps coordinates), each pairwise interaction would have a Schoener’s overlap value of 1, the total overlap value for the site would be 6, and the mean overlap per nest would be 1.5. If these nests were separated from each other by one standard deviation of the dispersal PDF, each pairwise overlap value would be 0.68, the total overlap for the site would be 4.08, and the mean overlap per nest would be 1.02. If these nests were separated from each other by two standard deviations, each pairwise overlap value would be 0.34, the total overlap for the site would be 2.04, and the mean overlap per nest would be 0.41. As nest locations become more distant from each other, the predicted index of overlap between each pair of nests would continue to diminish. Our analyses used the mean overlap index per nest as the population-wide contact metric.

***Section 1.5***

***Population-level behavioral time budget***

***Methods:***

To model larval movement, we need to know how much time they spend actively moving. From May 10 through June 4, 2020, we collected population-level movement behavior data from the Upton MA checkerspot population. These observations consisted of observing larval behavior at eight fixed observation points at the Upton population. Each observation was centered on a patch of *Plantago lanceolata* host plant and was conducted for 5 minutes, during which we scanned for larvae within 4m using binoculars. Larval behavior was classified as either moving, feeding, resting on a food plant, or resting on a non-food plant. These recurring observations took place on eight different sampling days, during which we observed a total of 2,783 larval behaviors. We examined whether behavioral budgets changed throughout larval development using linear models with observation date as a predictor and proportion of larva exhibiting a given behavior as the dependent variable.

***Results:***

Larvae spent an average of 23% (95% CI 20-26) of their time actively moving, 18% (95% CI 15-21) of their time actively feeding, 3% (95% CI 2-4) of their time sitting on plantain, and 55% (95% CI 51-59) of their time sitting on non-host plants adjacent to plantain. There were no significant differences in behavioral patterns as the growing season progressed (moving *F*_(1, 135)_ = 0.16, *P*= 0.69; feeding *F*_(1, 135)_ = 0.45, *P* = 0.51; sitting on plantain *F*_(1, 135)_ = 0.78, *P* = 0.38; sitting on other plants *F*_(1, 135)_ = 2.77, *P* = 0.38), indicating that these time budgets do not differ significantly between instars.

***Section 1.6***

***Duration of each instar***

***Methods:***

To estimate the amount of time a larva spends in each post-diapause instar, we monitored larvae that had been overwintered on Tufts campus. When 4^th^ instar larvae emerged from overwintering, we placed 10 larvae in each of six 15 cm plastic dishes kept them at room temperature. Each group of larvae was fed Plantago leaves daily, and we regularly measured their head capsule width and recorded signs of molting. When larvae pupated they were removed from the dish. We analyzed these data using a binomial glm describing the probability of each instar transition as a function of days since emergence from overwintering. To determine the average amount of time spent in each larval instar, we used the binomial model coefficients (*b)* and solved for the number of days that lead to a 50% transition probability: log(p/(1-p) = *b*_0_ + *b_1_**Days.

***Results:***

The mean time in the 4^th^, 5^th^ and 6^th^ instars were 15.2, 12.6, and 14.2 days respectively. The binomial glm model results for days to transition between the 4^th^ and 5^th^ instars, 5^th^ and 6^th^ instars, and 6^th^ instar to pupa are shown in Supplemental Table 3 and Supplemental Figure 3.

**Supplemental Table 3.** Results for instar transition binomial glm models.

| **Transition Probability Model** | **Factor** | **Coefficient** | **Std. Error** |
| --- | --- | --- | --- |
| 4^th^ to 5^th^ Instar | Intercept | -7.14 | 0.85 |
| 4^th^ to 5^th^ Instar | Days Since Emergence | 0.47 | 0.06 |
| 5^th^ to 6^th^ Instar | Intercept | -8.77 | 0.98 |
| 5^th^ to 6^th^ Instar | Days Since Emergence | 0.32 | 0.04 |
| 6^th^ Instar to Pupa | Intercept | -18.15 | 2.64 |
| 6^th^ Instar to Pupa | Days Since Emergence | 0.43 | 0.06 |

**
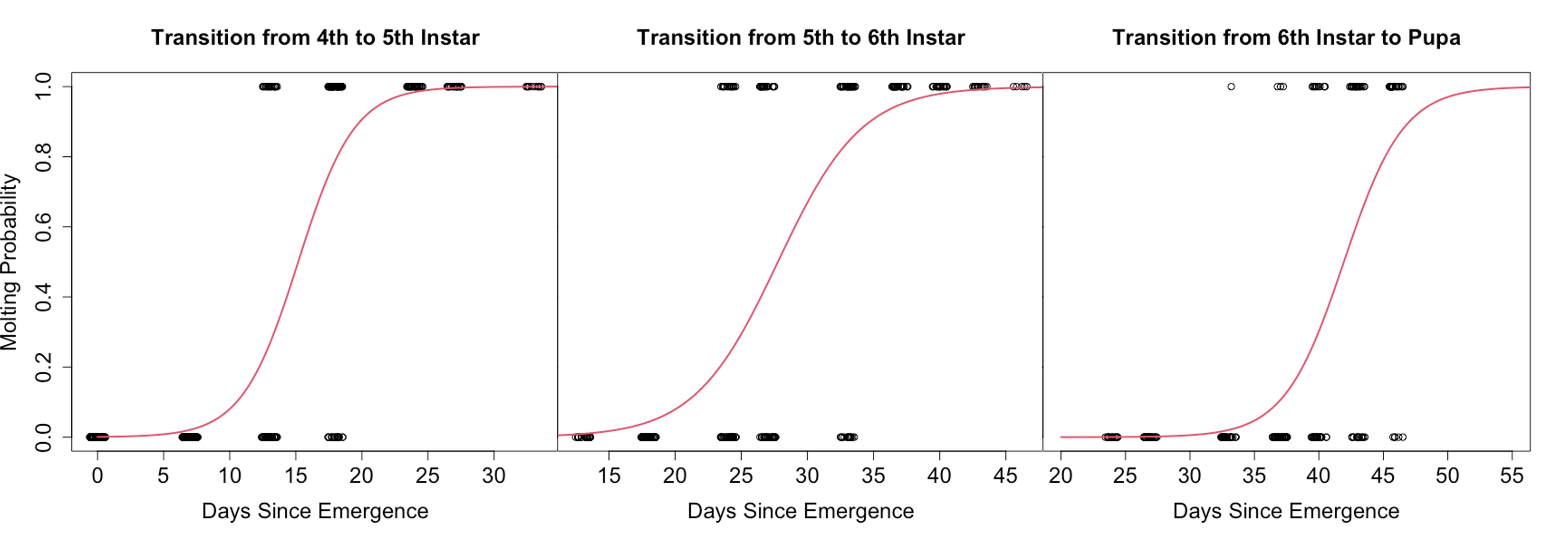
**

**Supplemental Figure 3.** The probability of larvae transitioning from 4^th^ to 5^th^ instar, 5^th^ to 6^th^ instar, and 6^th^ instar to pupae as a function of days since overwintering emergence. We solved for the number of days since emergence that the transition probability was 0.5 (dashed lines), and used these values to determine the mean time spent in each post-diapause larval instar. The mean time in the 4^th^, 5^th^ and 6^th^ instars were 15.2, 12.6, and 14.2 days respectively.

**Section 1.7**

***Relating Pre-diapause Nests to Total Population***

In this study, we used the number of larval nests as a proxy for population size. *E. phaeton* has discrete lifestages including egg, pre-diapause larvae in larval nests (instar 1-4), overwintering larvae (instar 4) in diapause, post-diapause foraging larvae (instar 4-6), pupae, and adult butterfly. Because there is little to no overlap between these lifestages, at any point in time the individuals represent the entire population. The post-diapause larval stage is believed to be the most important life stage for transmission of JcDV, and this study focused on the estimated contact occurring at this lifestage. However, post-diapause larvae are difficult to quantify in field surveys because they forage as individuals in dense vegetation, and molting prevents the use of mark/recapture methods. Therefore, we used pre-diapause larval nests as a proxy for the number of post-diapause larvae. The number of larvae in each nest is somewhat variable, ranging from 10-200 larvae per nest with a median of 50 (S. Figure 4). Approximately half of the larvae in pre-diapause nests survive the overwintering period and become post-diapause larvae (Brown et al., 2017b).

We conducted mark-recapture surveys of adult butterflies, and S. Figure 5 shows the relationship of larval nests in a given year and the number of adults from the same year (their parents) and the following year (the same individuals who survived to adulthood). Note that, because we collected data during a limited number of years, we have approximately 1.5 times as many observations for evaluating nest vs. abundance of their parents (N = 16 pairs) than for evaluating nest abundance vs. the resulting adult population in the next year (N = 11 pairs). Of course, the relationship between nests in one year and post-diapause caterpillars in the next is probably also tighter than the relationship between nests and the resulting adult population in the next year.

Overall these figures and associated regression results demonstrate a positive relationship between the number of larval nests and the number of adults in a population, but this relationship is complicated by the differential survival of larvae at different sites, as well as the number of host plants available from one year to the next. For example, in populations with high mortality in the overwintering, post-diapause, and pupal stages, the number of adults in year t+1 will be lower than expected relative to the number of larval nests in year t. High rates of larval mortality may result from the effects of winter temperature fluctuation (Abarca et al., 2019), parasitoids (Stamp 1980), or diseases including JcDV (Carson et al., in prep). Conversely, adult butterflies from sites with declining host plant availability will necessarily create fewer larval nests relative to their population size. This is especially true in years where post-diapause larvae consume the available host plants before pupation.


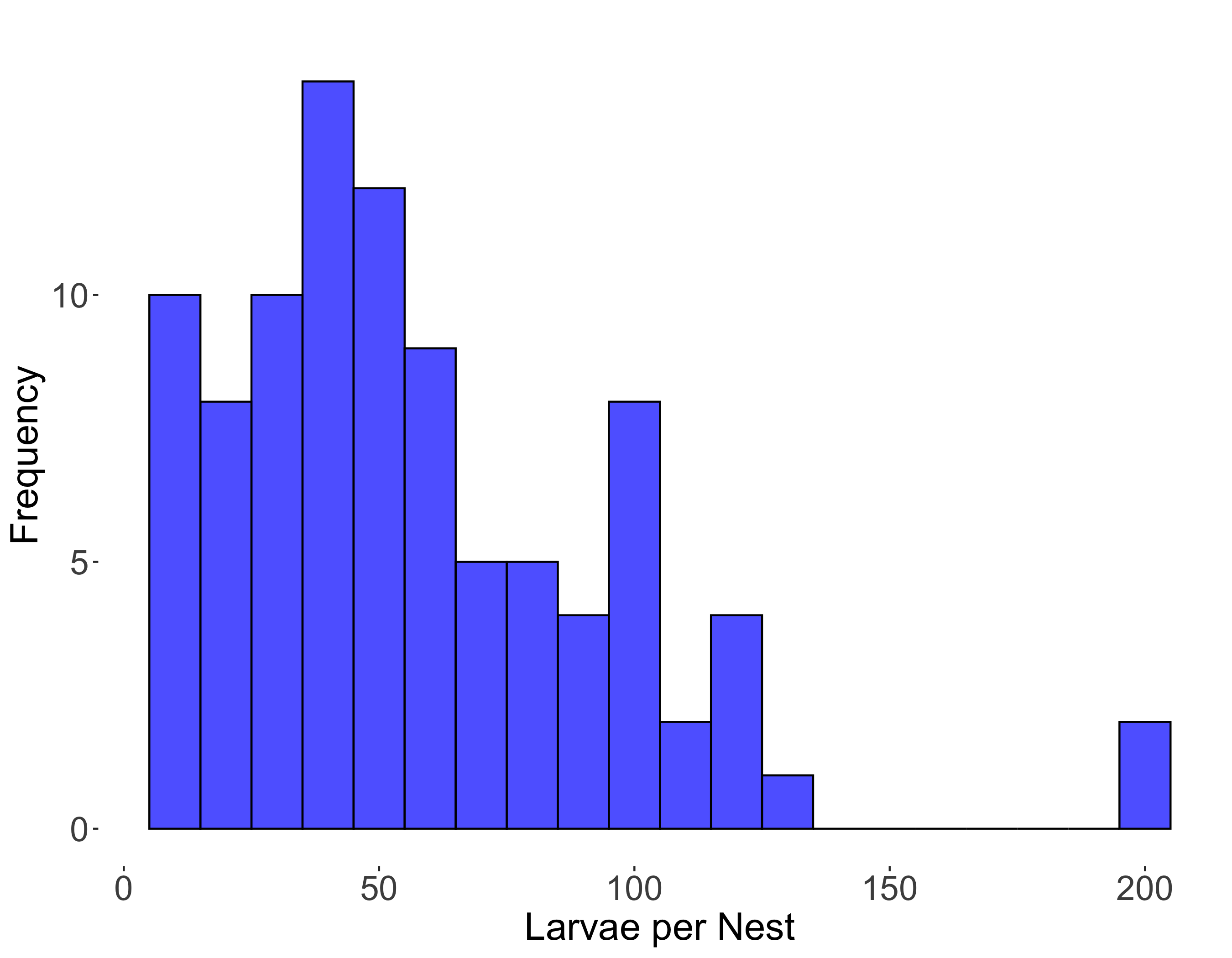


**Supplemental Figure 4.** Histogram of larval counts per nest in *Euphydryas phaeton*. Data from E. Crone and L. Brown (unpublished).


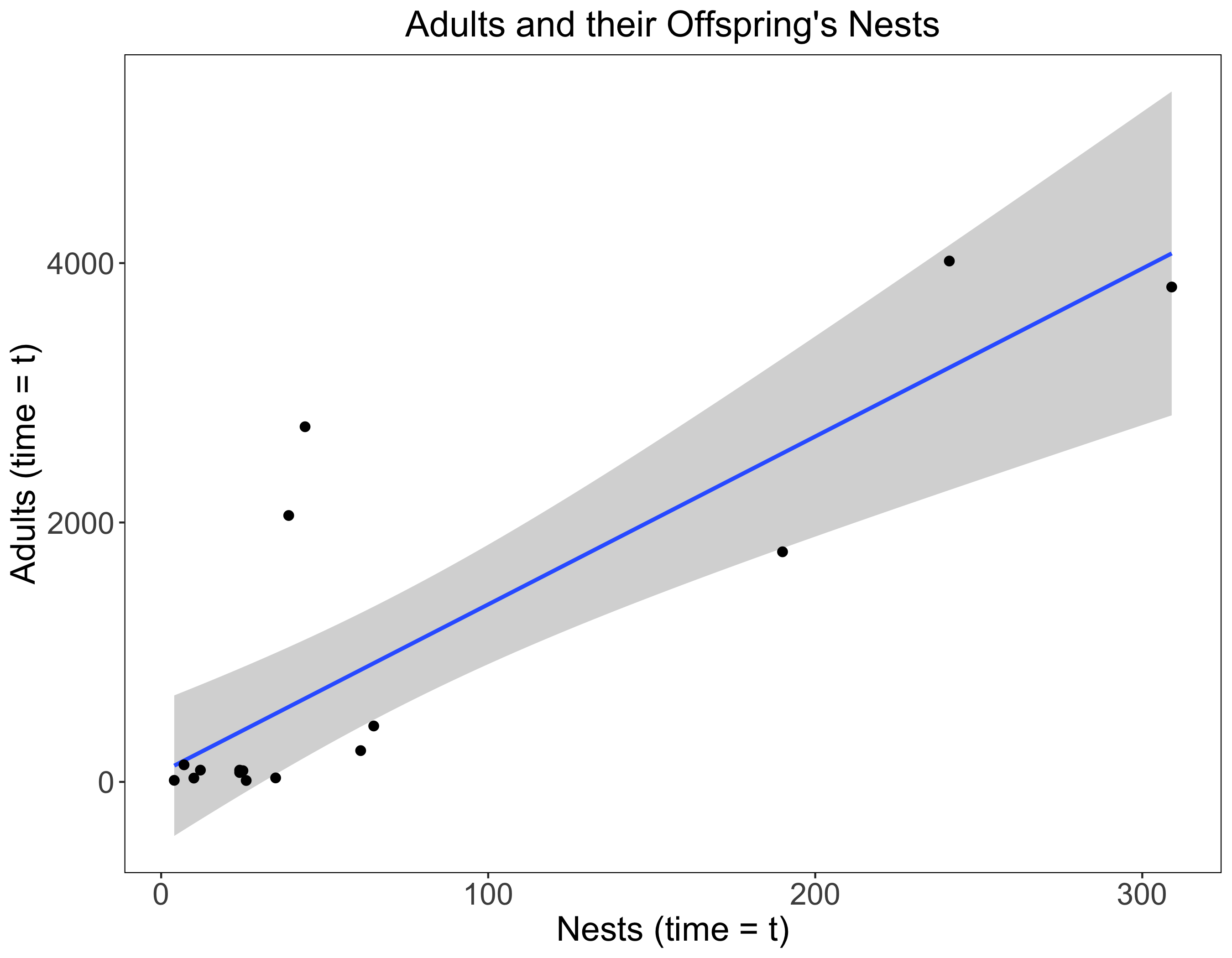

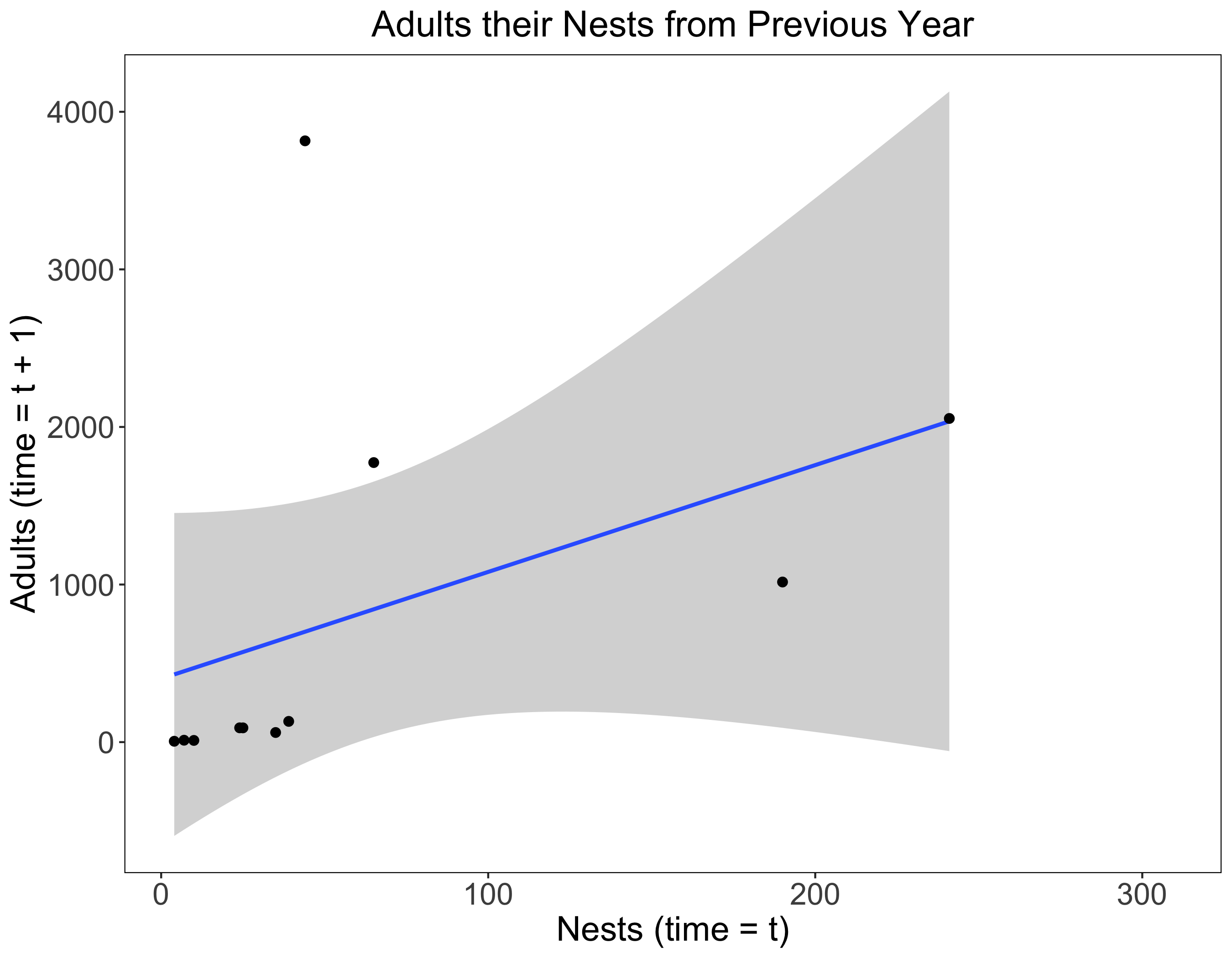


**a.**

**b.**

**Supplemental Figure 5.** **a)** The number of larval nests in a sampling year vs the number of adult butterflies in the same sampling year. In this case the sampled butterflies were the parents of the larvae in the nests
(R^2^ = .70, F= 32.26, df=1 and 14, p < .001) **b)** The number of larval nests in a sampling year vs the number of adult butterflies in the following sampling year. In this case the adult butterflies are the same individuals as those sampled in nests the previous year (R^2^ = .18, F= 2.02, df=1 and 9, p = 0.19).
